# Influence of Laser Shock Peening without Coating on Surface, Mechanical, Corrosion, and Tribological Characteristics of AA7075/h-BN Nanocomposites

**DOI:** 10.1021/acsomega.5c12393

**Published:** 2026-03-11

**Authors:** Sudheer Reddy Beyanagari, Vaira Vignesh Ramalingam, Jayakrishna Kandasamy, Katerina Skotnicova, Praveenkumar Kesavan

**Affiliations:** † Department of Mechanical Engineering, Amrita School of Engineering, 77649 Amrita Vishwa Vidyapeetham, Coimbatore 641112, India,; ‡ School of Mechanical Engineering, Vellore Institute of Technology, Vellore 632014, Tamil Nadu, India; § Faculty of Materials Science and Technology, VSB-Technical University of Ostrava, 17, listopadu 2172/15, Ostrava 70800, Czech Republic

## Abstract

AA7075 metal matrix composites have been widely used for their high strength-to-weight ratio. However, their relatively poor surface and tribological characteristics restrict their wider application in aerospace and automotive components. This study investigates the effect of laser shock peening without coating (LSPwC) on surface, mechanical, corrosion, and tribological properties of stir-squeeze cast AA7075, AA7075/0.5h-BN, and AA7075/1.0h-BN nanocomposites. LSPwC was carried out using a 1064 nm wavelength, 400 mJ laser pulse energy, 7.95 GW/cm^2^ power density, and 75% overlap. The LSPwC-treated specimens exhibited a significant improvement in surface hardness of ∼7% for as-cast AA7075 and ∼13% for AA7075/h-BN MMCs, primarily due to grain refinement, induced compressive residual stress (CRS), and intermetallic compound (IMC) strengthening. A reduction in surface roughness (*R*_a_) of ∼12% was observed in LSPwC-treated AA7075/h-BN in contrast to that in AA7075. Corrosion characteristics were enhanced through the formation of a stable and uniform passive oxide layer, thereby minimizing pitting corrosion in sacrificial anodic environments. The synergistic effect of h-BN and LSPwC enhanced surface wettability and tribological characteristics, resulting in a transfer of severe abrasive, adhesive, and delamination wear into predominantly milder adhesion and delamination wear. Overall, LSPwC-treated AA7075/1.0h-BN composites obtained the lowest SWR (0.1813 mm^3^/KN-m at RT and 0.8233 mm^3^/KN-m at 150 °C) and CoF (0.294 at RT and 0.314 at 150 °C).

## Graphical abstract

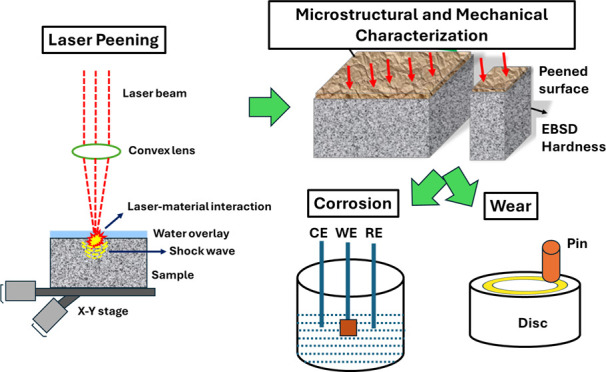

## 1 Introduction

Aluminum alloys (AAs), especially AA7075, are commonly used in aerospace, defense, automotive, and other industries due to their excellent mechanical performance.^1,2^ However, their poor tribological and corrosion behavior and surface degradation under cyclic loading limit their use in wear-intensive applications.^3–5^ To overcome these limitations, researchers have introduced ceramic and solid lubricant reinforcements such as Al_2_O_3_, B_4_C, h-BN, SiC, TiC, BN, Gr, MoS_2_, and so on into AA7075.^6,7^ The produced AA7075 metal matrix composites (MMCs) have exhibited better strength, hardness, and wear resistance compared to the base AA7075 alloy.^8,9^ Consequently, Al-MMCs have found applications in components such as pistons, cylinder liners, brake pads, brake drums, landing gear, and so on.^10,11^ Nevertheless, under higher loads and elevated temperatures, these components continue to experience severe wear, leading to reduced service life and increased maintenance costs.^12–14^

To further enhance the properties of AA7075 MMCs, several surface modification techniques, including cold rolling (CR),^15^ shot peening (SP),^16^ laser shock peening (LSP),^17–21^ ultrasonic shot peening (USP),^22^ surface mechanical attrition treatment (SMAT),^23^ coatings,^24,25^ friction stir processing,^26^ and others, have been explored. Among these, LSP is particularly effective due to its ability to introduce deep compressive residual stresses (CRS) (up to 1 mm) and refine grains without altering the substrate chemical composition.^27,28^ Laser shock peening without coating (LSPwC) simplifies the conventional LSP process by eliminating the ablative layer while employing controlled low-energy laser parameters to minimize thermal damage.^29–31^ Although LSPwC has demonstrated promising improvements in titanium and stainless-steel alloys,^32–36^ its application to Al-MMCs remains limited, thereby motivating the present investigation.

Recent studies have shown that LSP enhances surface hardness, corrosion resistance, and tribological behavior of AAs and their composites. LSPed Al7075/SiC/ZrO_2_ composites enhanced the surface hardness and microstructural integrity due to refined grains, uniform reinforcement distribution, and reduced *R*_a_.^37^ But detailed wear and corrosion evaluations were not adequately reported. In another study, powder metallurgy (PM)- and extrusion-processed Al6061/graphene nanocomposites exhibited superior mechanical properties compared to those of the base alloy. Upon LSP, there was further improvement in hardness (29.56%), tensile strength (24.55%), elongation (20.53%), and fatigue (50%) compared to the unpenned composite.^38^ A follow-up investigation on AA7075/graphene composites reported significant improvements in tensile strength (42%), yield strength (28%), and hardness (37%) compared to base AA7075. Upon LSP treatment, these properties were further improved by 10%, 30%, and 25%, respectively,.^39^ However, the interactive behavior of LSP with other reinforcement systems remains insufficiently explored.

Comparative surface modification studies on AA7075-T651 revealed that LSP produces a deeper hardened layer (up to ∼500 μm) and higher surface hardness than conventional SP and SSMT.^40^ Subsequent optimization studies highlighted the strong influence of laser energy, overlap ratio, and pulse parameters on residual stress and fatigue performance.^41^ Low-energy LSP-treated AA7075-T651 exhibits a 27% improvement in corrosion resistance, attributed to a reduction in microstructural surface cracks and the uniform formation of nanograins, which contribute to an enhanced surface structure.^42^ In another study, a low pulse energy of 400 mJ with a spot diameter of 1 mm and 6 repetitions exhibited 3.7 times higher hardness, improved roughness, and 65% greater wear resistance compared to the untreated sample.^43^ A similar process resulted in a 29% increase in microhardness and improved resistance to fatigue and wear in the AA7075.^44^ At a temperature of 300 °C, a significant decrease in the CTE on the nanocrystalline surface structure of the Al7075/TiC composites is observed after LSP treatment.^45^ This is mainly attributed to an increase in the dislocation density, grain refinement, and phase transformation.

The mechanical and corrosion characteristics of AA7050 subjected to nanosecond LSP (NLSP) with an overlayer and femtosecond LSP (FLSP) without an overlayer are compared. Results indicated improved corrosion resistance under both conditions due to maximum hardness and high CRS near the peened surface. NLSP demonstrates a more sustained effect, with hardness and residual stress decreasing gradually with depth. Whereas, FLSPs’ effects are more localized to the surface due to rapid shock wave attenuation.^46^ Experimental and simulation approaches are employed to investigate the impact of LSP on residual stress behavior of AA7075/TiC composites, in comparison with the unreinforced alloy. The results found that TiC reinforcing particles significantly enhanced the strengthening effect of LSP in the AA7075 composites. The TiC NPs controlled the shock wave propagation by absorbing TRS and increasing the CRS near the surface. Also, this phenomenon helped to modify the typical compressive-tensile-compressive stress distribution in the AA7075/TiC composite.^47^

Despite extensive studies on LSP of AA7075 and its composites, the synergistic effect of LSPwC and solid lubricant-based reinforcements on tribological (wear and friction) behavior of AA7075 under room and elevated temperatures has not been systematically investigated. In particular, the role of h-BN nanoparticles in governing shock-wave interactions, microstructural evolution, and wear-friction mechanisms in AA7075 remains unclear. Therefore, this study aims to bridge this research gap by investigating the influence of LSPwC on the microstructural, surface, corrosion, and tribological characteristics of stir-squeeze cast AA7075/h-BN nanocomposites.

## 2 Materials and Methods

### 2.1 Selection of Materials

In this study, AA7075 was chosen as the matrix and h-BN NPs (particle size ∼100 nm) as the reinforcement. The AA7075/h-BN nanocomposites were prepared in the stir-squeeze casting (SSC) technique by incorporating 0, 0.5, and 1.0 wt % of h-BN NPs. Approximately 1.5 kg of AA7075 was melted in a graphite crucible at 850 °C, attached to an SSC setup. A hexachloroethane (C_2_Cl_6_) tablet was subsequently introduced into the molten bath to remove impurities (dissolved gases and surface slag). The metal was stirred at 300 rpm for 5 min using a graphite-coated hydraulic stirrer. Preheated h-BN nanoparticles (at 500 °C) were gradually introduced along with a small quantity of preheated Mg to improve wettability and bonding with the Al matrix. The die and pouring path were preheated to 350 °C to minimize thermal gradients. After pouring, a squeeze pressure of 150 MPa was applied for 60 s, followed by a delay of ∼10 s to allow for solidification. This process enhanced the castability, particle distribution, and reduced porosity in the produced AA7075/h-BN composites. The produced AA7075 (as-cast), AA7075/0.5h-BN, and AA7075/1.0h-BN were chosen as substrates for further studies. The detailed casting procedures and reasons for opting for these casting parameters were discussed in our earlier studies.^48–50^

### 2.2 Laser Shock Peening without Coating (LSPwC) on AA7075/h-BN Nanocomposites

Laser shock peening (LSP) was performed on fine polished AA7075/h-BN nanocomposites using a Q-switched Nd/YAG laser operated at a fundamental wavelength of 1064 nm, constant pulse energy of 400 mJ, power density of 7.95 GW/cm^2^, repetition rate of 10 Hz, and pulse duration of 10 ns. The laser beam was reflected using a dichroic mirror positioned at 45° and focused through a biconvex lens with a focal length of 758 mm, resulting in a beam diameter of 0.8 mm at the sample surface with a 75% overlap. Existing literature reports that low-to-moderate pulse energies (≤500 mJ) induce shock pressures beyond the Hugoniot Elastic Limit (HEL), thereby generating stable CRS and grain refinement while avoiding thermal damage under LSPwC conditions.^31,40,42,43^ The sample was mounted on an X–Y translation stage driven by servo motors with a resolution of 0.1 μm and controlled via multi-CNC software. A thin water layer 1 mm in thickness, sourced from treated tap water, served as a confining medium. A 20 mm × 20 mm × 10 mm area was exposed for peening to characterize the morphological and mechanical characteristics. Cylindrical samples (6 mm diameter × 30 mm length) were processed along both the longitudinal (*X*-axis) and radial (*A*-axis) directions for friction and wear characteristics. The peening process began at the starting point, as shown in the figure, where the sample was treated along the 30 mm axial length, followed by a 5.0° rotation around the *A*-axis to maintain the 75% overlap. This LSPwC with controlled rotation was continued until the completion of one full rotation (360°). Two times peening was performed on the sample to ensure uniform treatment. Figure 1 illustrates the schematic procedure of LSPwC along with the designated peened area.

**Figure 1:**
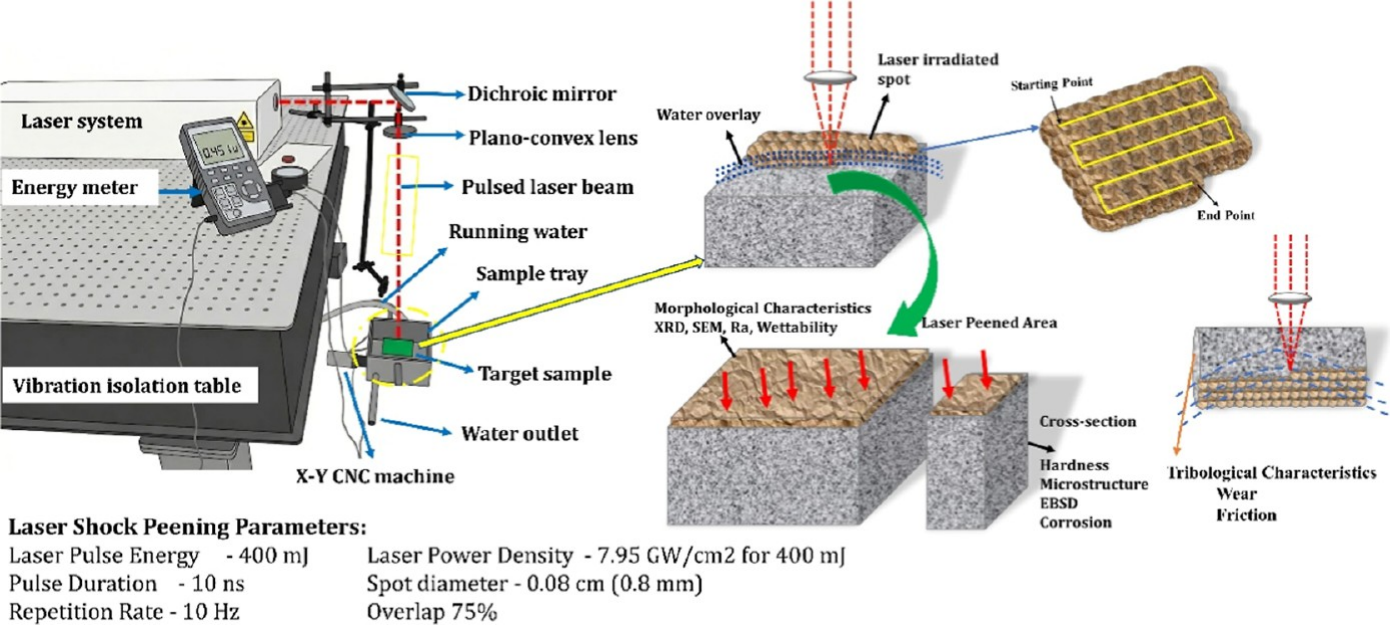
LSP_W_C setup used for surface modification of AA7075/h-BN nanocomposites.

### 2.3 Testing of the Composites

The tribological and mechanical characteristics of the produced samples were measured according to ASTM standards, including the wear rate, coefficient of friction, and hardness. Surface characteristics, such as microstructure, morphology, phase composition, roughness, wettability, surface energy, and corrosion characteristics, were examined.

#### 2.3.1 Morphological Characteristics

LSPwC and un-LSPwC AA7075/h-BN nanocomposite samples were polished (cross-sectionally for LSPwC) using emery papers (600 to 2000 μm), followed by fine polishing with diamond paste and water on a low-speed disc-polishing machine. Keller’s reagent was used for etching (10–20 s) and cleaned with distilled water. Surface morphology was examined using an optical microscope (Olympus BX 61) and FE-SEM (Thermo Fisher FEI-Quanta 250 FEG). Phase analysis was carried out using XRD (Bruker D8-Advance) over a 10–90° 2θ range with a step size of 0.01708.

#### 2.3.2 Hardness

Cross-sectional hardness LSPwC AA7075/h-BN composites were compared with the unpeened AA7075/h-BN nanocomposite using a Vickers hardness tester (Matsuzawa, Japan) according to ASTM E-381 and IS 1501:2002 standards. The test was conducted at room temperature with a load of 200 g and a dwell time of 10 s. Average hardness was calculated from 8 readings at 150 μm intervals, and the standard deviation was used for error bars.

#### 2.3.3 Surface Roughness

Surface roughness of unpeened and LSPwC AA7075/h-BN nanocomposite was measured using a Mahr MarSurf GD 120 surface roughness tester. The arithmetic average roughness (*R*_a_) of the profile height over the evaluation length was recorded, and the results are discussed in Section 3. Further, 3D surface profiles and *R*_a_ were measured using the Keyence digital microscope (VHX-7000N) for the LSPwC AA7075/h-BN nanocomposite.

#### 2.3.4 Contact Angle Measurement

The contact angle was measured by observing the intersection angle at the two ends of the liquid/solid interfaces. In this study, a goniometer (Phoenix 300, SEO, Korea) was used to measure the contact angle using the sessile drop technique. Distilled (DI) water was used as the test liquid/solvent. A syringe was employed to carefully dispense and place a drop of DI water on the surface of the AA7075/h-BN nanocomposite. Then, contact angles were captured and recorded with a high-resolution camera attached to the device. Also, the surface energy (*E*_s_) was calculated as per eq 1.

1 $$E_{s}=\gamma \cos\phi$$

Where ϕ is the contact angle and γ is the surface tension of distilled water (72.8 mJ/m^2^).

#### 2.3.5 Corrosion Characteristics

The corrosion characteristics of unpeened and LSPwC AA7075/h-BN nanocomposite were measured using a standard three-electrode electrochemical workstation (CH Instruments, Model: 680 Amp Booster) in a 3.5% NaCl solution (electrolyte). The setup included a working electrode (AA7075/h-BN), a reference electrode (calomel), and a counter electrode (platinum). Finely polished specimens were masked to expose a 1 mm^2^ surface area to the electrolyte solution as per the ASTM E3-11 standards. Open circuit potential (Eocp) was stabilized through stabilization of potential within ±10 mV (∼6 h). Subsequently, the specimen was subjected to a potentiodynamic polarization scan between −1.2 V and −0.2 V with respect to Eocp. The corrosion potential, *E* corr, was identified at the intersection of net anodic (β_a_) reactions equal cathodic (β_c_) Tafel slope. The Tafel region between *E* corr ±20 mV was extrapolated to determine corrosion current *I* corr (*A*). Linear polarization resistance (LPR (*Q*)) and corrosion resistance (CR) were determined using eqs 2 and 3.

2 $$LPR\left(Q\right)=\frac{\beta_{a}\times \beta_{c}}{2.3\times \left(\beta_{a}+\beta_{c}\right)}\times \frac{1}{i_{corr}}$$

3 $$CR=\frac{i_{corr}\times M_{w}}{Z\times A\times \rho }$$

Where *M*_w_—molar weight of the specimen, *Z*—number of electrons of the specimen, *A*—exposed area, and ρ—density of the specimen.

The breakdown potential *E*_b_ (V) was identified in the anodic curve, where a sudden surge in the current was observed. Three samples from each specimen were tested, and the average was reported from the ECC.

#### 2.3.6 Tribological Characteristics for LSP_W_C and Un-LSP_W_C AA7075/h-BN Nanocomposites

The friction and wear behavior of AA7075/h-BN nanocomposites was evaluated using a pin-on-disc tribometer as per ASTM G-99 standards. Both LSPwC and unpeened pins (6 mm diameter, 30 mm height) were tested against an EN31 steel disc. The reason for choosing EN-31 steel disc as a counter material is owing to its higher hardness (62 HRC), elastic modulus (210 GPa), and excellent wear resistance, while the surface roughness of the disc was 0.3 μm.^48–50^ The experiments were performed under an applied load of 40 N, a sliding velocity of 2 m/s, and a sliding distance of 1000 m at room temperature (30 °C) and elevated temperature (150 °C). The counter disc surface was cleaned with acetone before each test run to avoid contamination. Pin masses were measured before and after the test using a high-precision electronic balance (0.0001 g accuracy). Specific wear rates (SWR) and CoF were determined using standard eqs 4 and 5, respectively. The worn-out surface morphology of the AA7075/h-BN pins was analyzed using SEM.

4 $$SWR=\frac{\Delta m}{\rho \times D\times L}\left(\frac{{mm}^{3}}{N-m}\right)$$

Where Δ*m*—mass loss of the sample during the test (g), ρ—density of the sample (g/cm^3^), *D*—sliding distance (*m*), and *L*—applied load (N).

5 $$CoF=\frac{F_{f}}{F_{N}}$$

Where, *F*_f_—frictional force (N) and *F*_N_—applied force (load) (N).

## 3 Results and Discussion

### 3.1 Morphological Characteristics of LSPwC AA7075/h-BN Nanocomposites

The surface morphology of AA7075/h-BN nanocomposites before and after LSPwC is analyzed using an optical microscope and FE-SEM analysis, as shown in Figures 2 and 3. The OM micrographs reveal that the inclusion of h-BN nanoparticles results in finer and more equiaxed grain boundaries compared to the coarse dendritic grains visualized in the as-cast AA7075. Where the h-BN NPs act as effective nucleation sites during solidification and restricted dendritic growth, leading to a significant reduction in grain size (ref Figures 2 and 3 of unpeened microstructure). The cross-sectional microstructure of LSPwC AA7075/h-BN MMCs reveals further grain refinement, with closely packed and uniformly distributed equiaxed grains. This grain refinement is attributed to repeated high-strain-rate plastic deformation induced by laser shock waves (ref Figure 2 LSPwC microstructure). Intermetallic precipitates such as MgZn_2_ and Al_2_CuMg phases appeared inside and along the grain boundaries. However, during LSPwC, water acts as the cooling medium, which quickly dissipates heat and limits excessive thermal effects, leading to rapid solidification and limiting the formation of the closed grain structure.^31,51,52^

**Figure 2:**
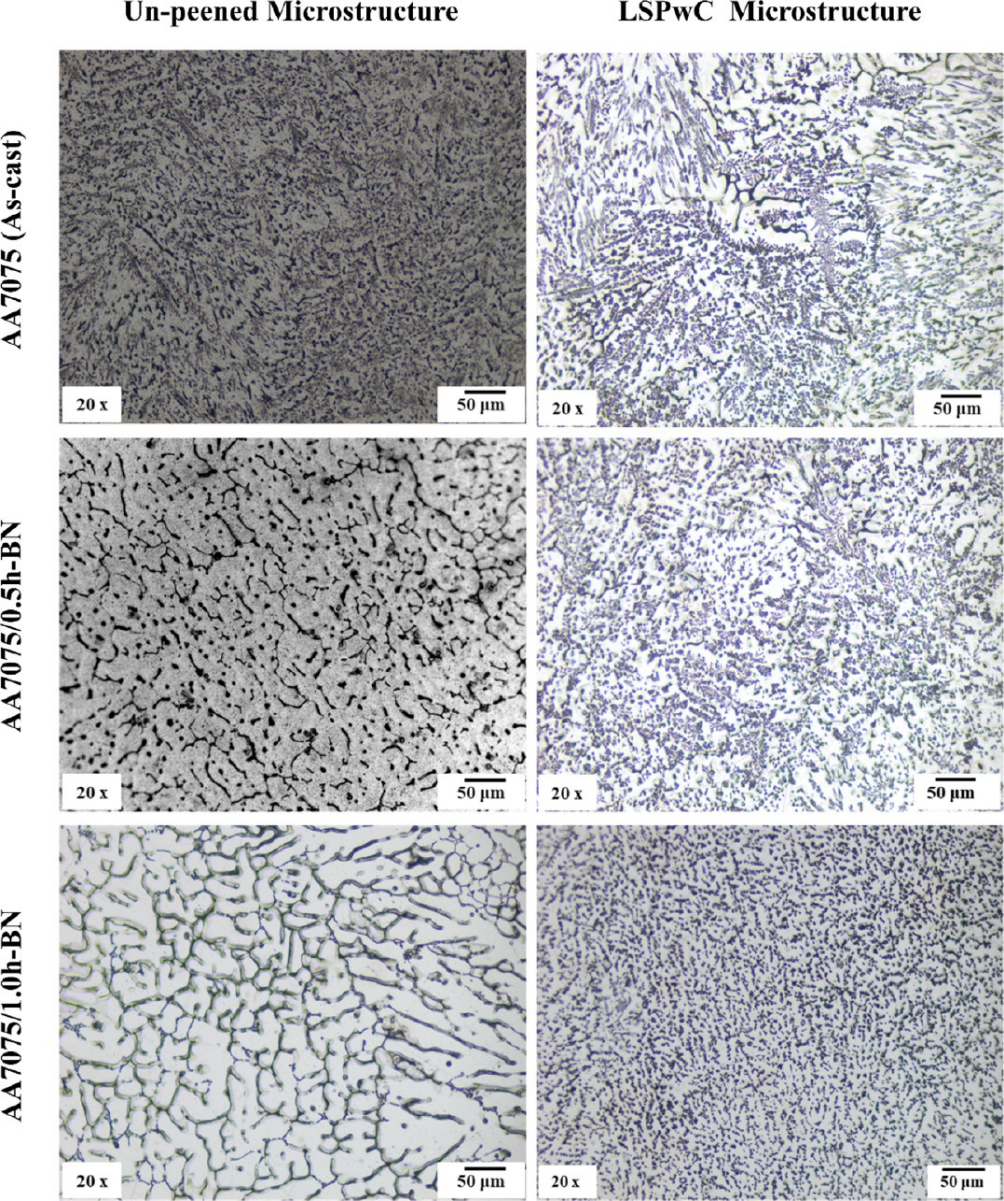
Optical microstructural characteristics of LSP_W_C AA7075/h-BN nanocomposites.

**Figure 3:**
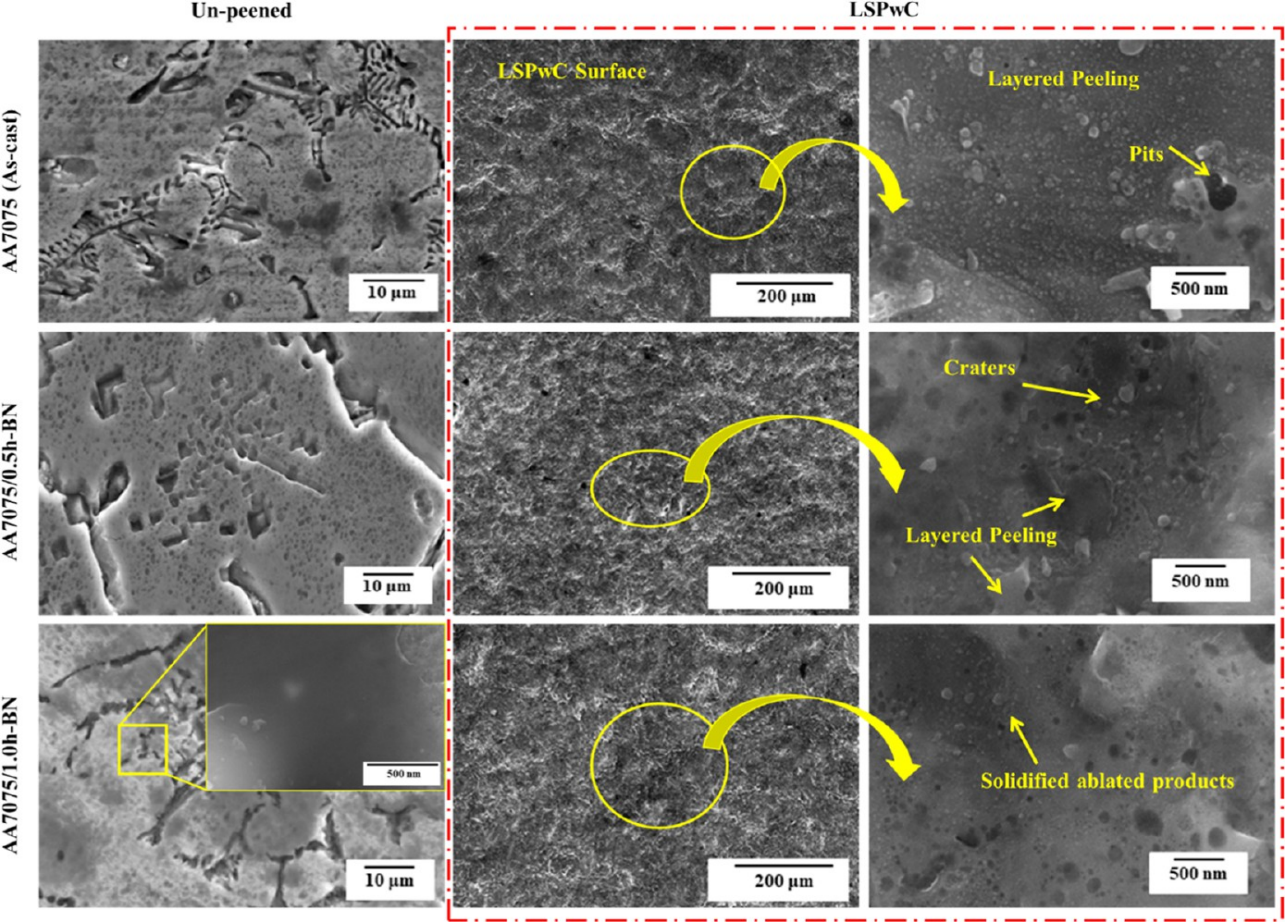
Surface morphological characteristics of LSP_W_C AA7075/h-BN nanocomposites.

The surface topography of post-LSPwC AA7075/h-BN composites, shown in Figure 3, exhibits numerous indentation features like craters that widened and deepened, as well as localized surface undulations, which lead to noticeable roughness. These features form due to the direct interaction of laser pulses with the surface, causing localized melting and subsequent resolidification at high temperatures and strain rates. This process increases dislocation density and promotes grain refinement. Despite the severe thermo-mechanical loading, no surface or subsurface microcracks are observed, even at higher magnifications. This crack-free surface is attributed to controlled laser energy input, progressive peening, and the presence of h-BN nanoparticles, which enhance load distribution and resist localized deformation during peening.

Elemental mapping (EM) and EDS analysis of unpeened and LSPwC AA7075/h-BN nanocomposites are shown in Figure 4. It is confirmed that there is a uniform distribution of Zn, Mg, Cu, B, and N elements in the matrix material (Al alloy). A noticeable increase in the content of O is observed in the LSPwC-treated samples. This is mainly attributed to the formation of a thin oxide layer due to the decomposition of the water confinement layer under laser-induced plasma and the intense electric field of the laser pulses, as supported by earlier studies.^29,40,53^ After LSPwC, the stable presence of h-BN nanoparticles and intermetallic phases of MgZn2 and Al2CuMg, within the α-Al matrix, is observed. This further contributes to enhanced microhardness and surface integrity of LSPwC AA7075/h-BN nanocomposites.

**Figure 4:**
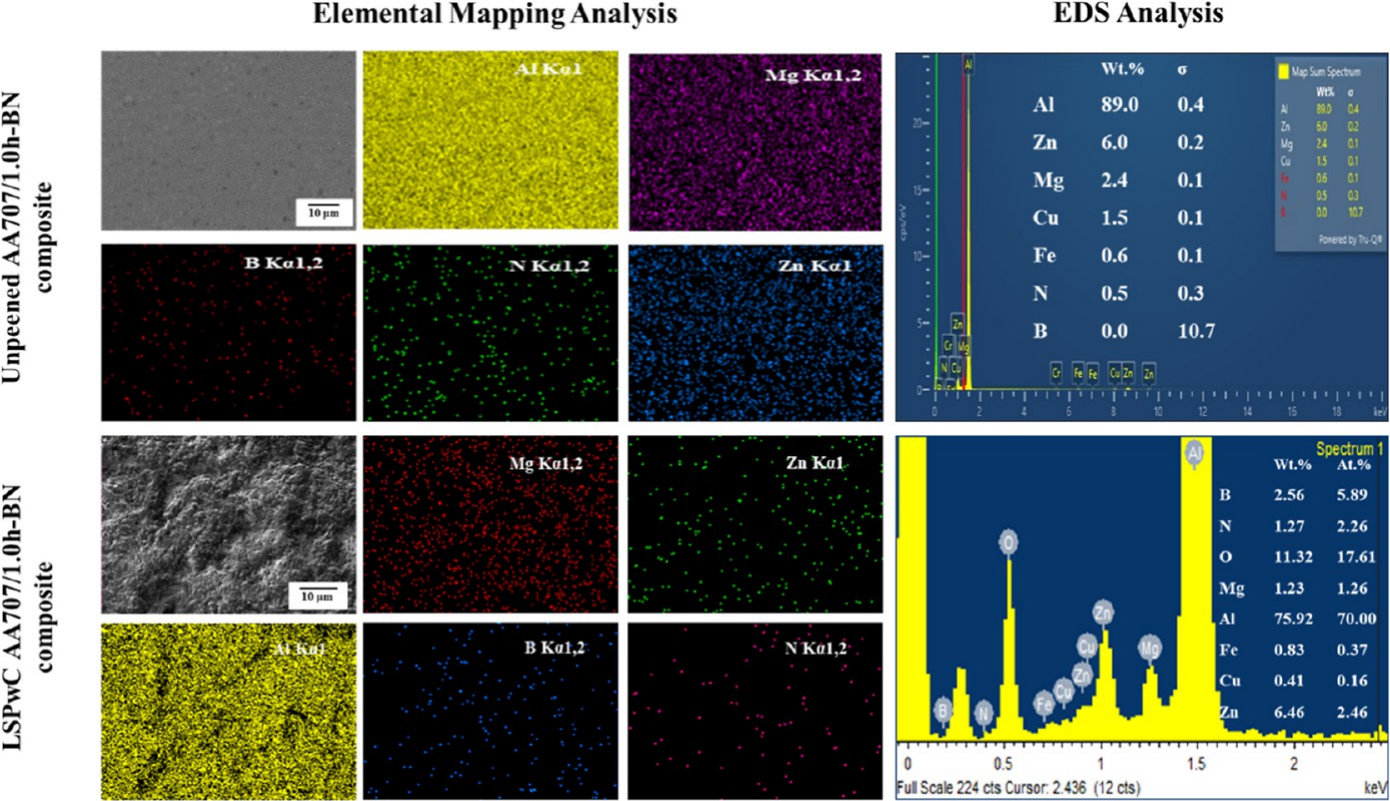
Elemental Mapping and EDS analysis of the pre- and post-LSPwC-treated AA7075/h-BN nanocomposites.

The texture analysis of AA7075/h-BN and LSPwC AA7075/h-BN nanocomposites is shown in Figures 5 and 6. From the IQ maps, a rough and heterogeneous surface was observed on as-cast AA7075. The formation of such features was mainly due to the uneven grain structure. Inclusion of h-BN NPs in AA7075 helps to improve the grain structure (grain refinement) and reduces surface heterogeneity of AA7075/h-BN nanocomposites. Where h-BN NPs serve as a nucleation site and hinder the grain expansion during solidification due to the Zener pinning effect. Further, LSPwC significantly refined the microstructure of all specimens due to the bombardment of high-energy laser pulses, which induced plastic deformation and promoted DRX. The LSPwC AA7075/1.0h-BN nanocomposite specimen exhibits optimal crystallographic quality attributed to the presence of h-BN NPs (1.0 wt %), which promote effective grain boundary pinning and DRX with LSPwC. The PFs’ and IPFs’ map reveals large and randomly oriented grains in the AA7075 (as-cast) sample, which are all slightly refined (mild texture), oriented uniformly, and texture development is attributed to restricted grain boundary motion with the presence of h-BN NPs in AA7075. These grains were further split/fragmented and then oriented uniformly with a sharpened texture due to the realignment of grain orientations (because of laser shock-induced plastic deformation), resulting in uniform crystallographic texture, particularly for the LSPwC AA7075/1.0h-BN nanocomposite. The dislocation densities of AA7075/h-BN nanocomposites gradually decreased with the inclusion of h-BN NPs, as seen in GND maps. The h-BN NPs effectively restricted the motion of dislocation and enhanced load transfer between the AA7075 matrix and h-BN NPs’ reinforcements compared with AA7075 (as-cast). Furthermore, a substantial reduction in GNDs is observed after LSPwC, indicating pronounced strain hardening, followed by dislocation annihilation, pile-up, and rearrangement. These mechanisms promote dynamic recovery and recrystallization, leading to a refined microstructure characterized by subgrain boundaries, additional precipitates, and finer grains.^54^

**Figure 5:**
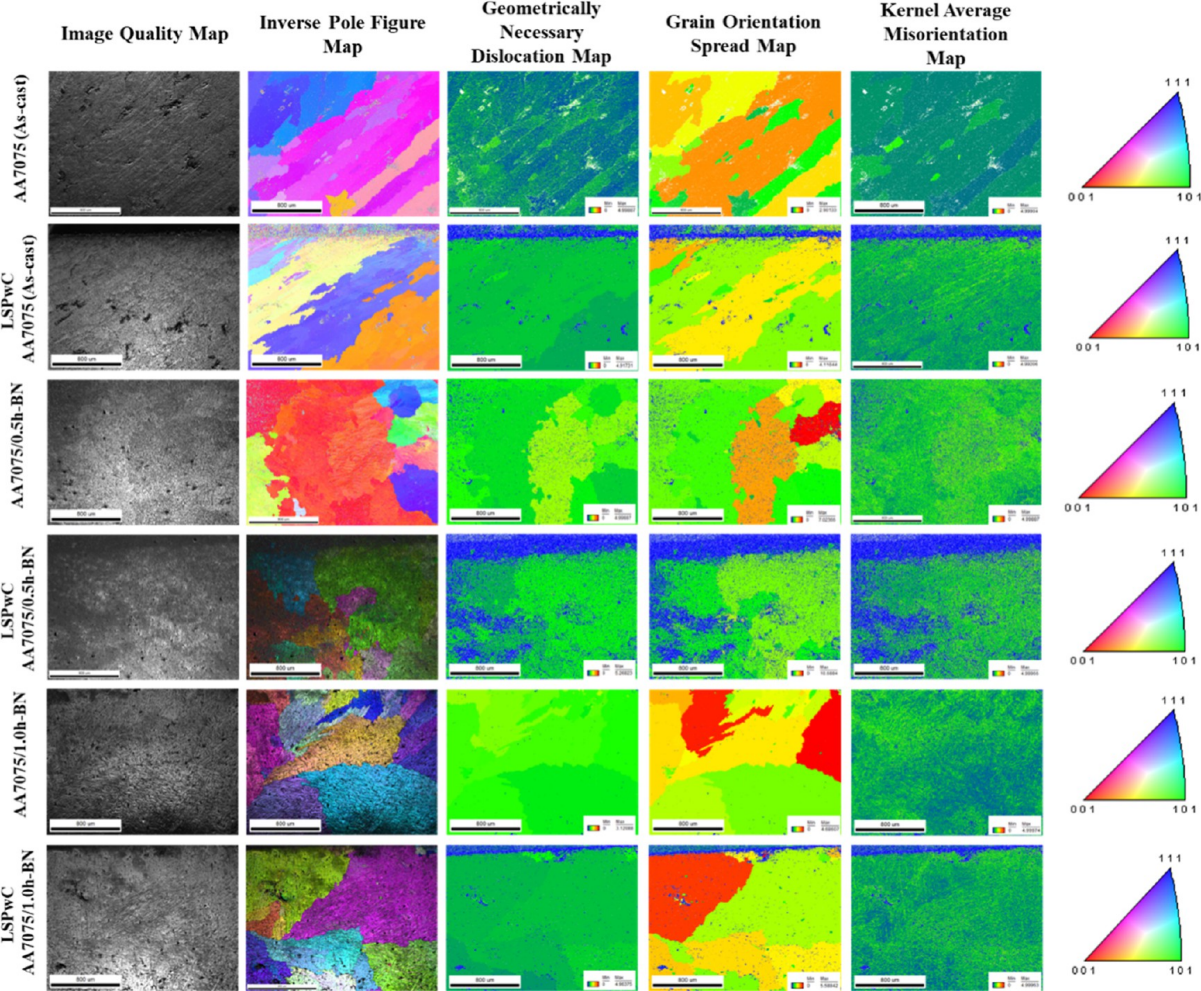
EBSD microstructural analysis of LSPwC AA7075/h-BN nanocomposites.

**Figure 6:**
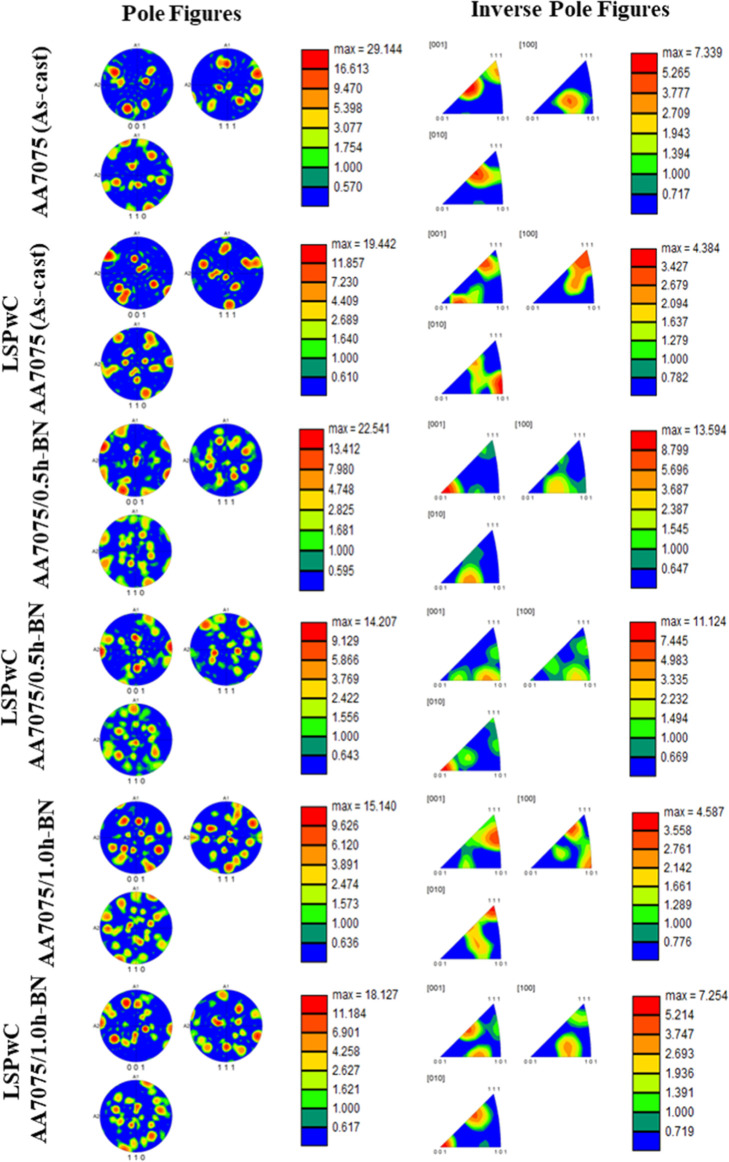
Pole figures (PFs) (A1 indicates RD and A2 indicates TD) and inverse pole figures (IPFs) of LSPwC AA7075/h-BN nanocomposites.

The AA7075 (as-cast) specimen exhibits a larger texture with high misorientations, as revealed from the GOS map. This was attributed to local strain accumulation, and inhomogeneous plastic deformation occurred in AA7075 (as-cast). The addition of h-BN NPs acts as a barrier toward dislocation movement and reduces these misorientations by stabilizing the grain boundaries. Post LSPwC reveals a decreased GOS value across all the samples, indicating more uniform strain distribution and enhanced structural homogeneity in the AA7075/h-BN nanocomposites. Similarly, KAM maps show high local misorientation in the AA7075 (as-cast) due to a high level of residual plastic strain, which was reduced after the incorporation of h-BN NPs. Further, LSPwC relieves internal stresses and reduces stored internal energy, resulting in lowering KAM values and enhancing lattice stability of the AA7075/h-BN nanocomposites.

The PFs and IPFs of unpeened and LSPwC-treated AA7075/h-BN nanocomposites are shown in Figure 6. The unpeened AA7075 exhibits strong preferred orientations due to the solidification-induced texture. The addition of h-BN NPs weakens this texture by promoting particle-stimulated nucleation and random grain orientation. After LSPwC, both pole and inverse pole figures display diffused intensity, indicating texture randomization caused by high-strain-rate plastic deformation and dynamic recrystallization. The repeated shock loading refines grains and increases the dislocation density, leading to orientation breakup. LSPwC AA7075/1.0h-BN shows the most homogeneous texture, confirming the synergistic effect of reinforcement and laser peening. A similar kind of texture analysis was reported in earlier studies.^38,39^

### 3.2 Phase Analysis of LSPwC AA7075/h-BN Nanocomposites

Different crystalline planes were observed in the XRD patterns of the unpeened and LSPwC AA7075/h-BN MMCs, as shown in Figure 4. The obtained crystalline planes were validated by using various JCPDS files and X’pert High Score (XPS) software. It was identified that the major phases correspond to the Al(111), (200), (220), and (113) planes, which exhibit noticeably intense intensities in all of the specimens. Precipitates like S­(MgAlCu) and η­(MgZn_*x*_) intermetallic compounds were also detected at lower intensities in both unpeened and LSPwC AA7075/h-BN composites. This confirms the polycrystalline nature of the phase structure for all of the specimens. Observation of the main Al phase reflections revealed largely consistent peak positions, implying that the residual stress generated by LSPwC did not measurably alter the crystal’s lattice parameter. However, a closer observation of XRD patterns (Figure 7) reveals that the peaks of h-BN are present in both unpeened AA7075/h-BN and LSPwC AA7075/h-BN composites. While in the LSPwC AA7075/h-BN composites, peaks are shifted slightly toward the left side. This peak shift indicates the sign of CRS due to induced LSPwC. Additionally, peak broadening at full width at half-maximum (fwhm) was observed mainly in the LSPwC specimens, which signifies an increase in the dislocation density due to the plastic deformation caused by LSPwC. The enhanced crystalline structure was also attributed to the existence of h-BN NPs. The variations in lattice strain/distortion, peak position, crystalline size, dislocation density, and phase transformation changes were the primary reasons for the enhancement in hardness and the induction of CRS after peening. Also, in the unpeened reinforced samples, a slight development of CRS occurred due to the high squeeze pressure applied during the casting process. The CRS was further increased by peening due to the higher shock wave pressure generated by laser pulses on the surface of the AA7075/h-BN nanocomposites during LSPwC.

**Figure 7:**
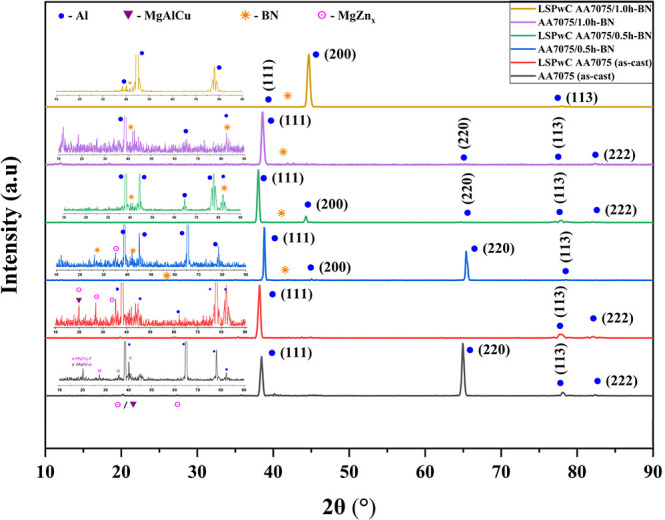
Phase analysis of LSPwC AA7075/h-BN nanocomposites.

### 3.3 Surface Roughness of LSPwC AA7075/h-BN Nanocomposites

The average *R*_a_ of all unpeened AA7075/h-BN MMCs was grounded to 0.25–0.5 μm before peening. The surface roughness (*R*_a_) of LSPwC AA7075/h-BN MMCs is shown in Figure 8. It was common that after peening, *R*_a_ will significantly increase due to the direct laser pulses’ ablation on the sample surface without a sacrificial layer that was induced on the surface and thereby craters/indentations were formed on the surface (ref Figure 3 shows the morphology of LSPwC AA7075/h-BN nanocomposites). It was evident that the AA7075 (as-cast) exhibited the highest *R*_a_ value ∼2.3665 μm as measured from the Mahr MarSurf GD 120 surface roughness tester (2D roughness measurement), and ∼52 μm overall surface roughness (*S*_a_) was obtained from the 3D profilometer. From the 3D surface scans, rougher, deeper valleys and peaks were observed, indicating an uneven surface resulting in higher surface roughness. However, lower roughness was observed with LSPwC-treated AA7075/h-BN samples than with the LSPwC-treated AA7075 (as-cast). For LSPwC-treated AA7075/0.5h-BN composites, the *R*_a_ was reduced to ∼2.2954 μm (2D) and *S*_a_ to ∼40 μm (3D). Similarly, *R*_a_ was reduced to ∼2.2092 μm (2D) and *S*_a_ to ∼30 μm (3D) for AA7075/1.0h-BN MMCs. The main reason was that the presence of white graphene (h-BN) NPs were strongly bonded with the AA7075 matrix and acted as a sacrificial layer to produce the least plastic deformation during peening. Further, with the presence of h-BN particles, the surface becomes more uniform with reduced peaks and valleys because it acts as a barrier for excessive deformation. Overall, after LSPwC, *R*_a_ increased for AA7075/h-BN composites, which was also reported in many studies on cast AAs, Ti alloys, and steels.^52,55,56^ However, the *R*_a_ increased as compared to the unpeened specimens, but due to the presence of 2D h-BN NPs certainly influenced the laser-ablated regions and depressed layer by transferring the laser shock wave compression on the surface due to its load-bearing nature. Thus, it is proved by experimentation that the minimum roughness, i.e., a better surface condition leading to enhanced tribological behavior, can be achieved with AA7075/1.0h-BN by the addition of solid lubricants in comparison to unpeened AA7075 (as-cast).

**Figure 8:**
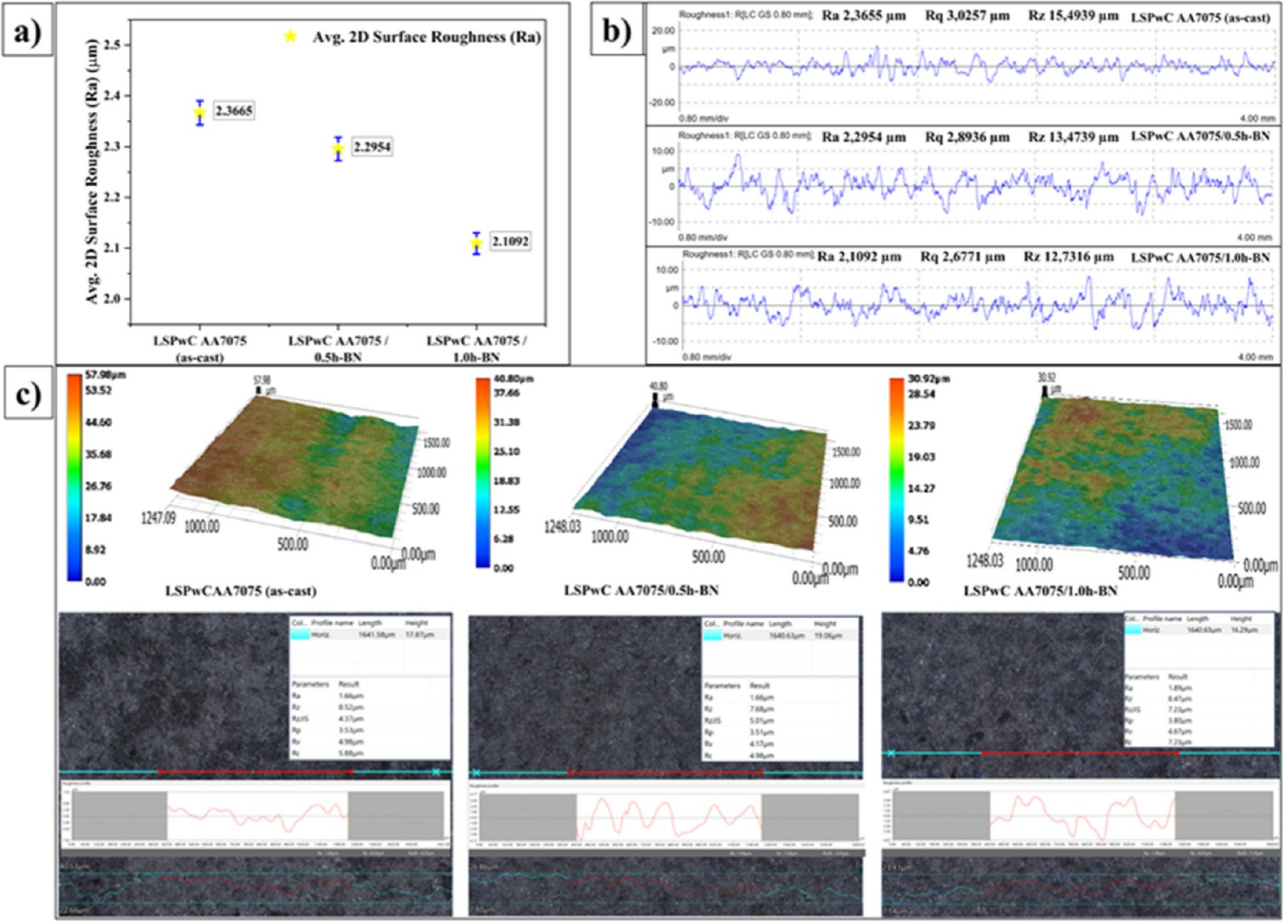
Roughness of LSP_W_C AA7075/h-BN nanocomposites. (a) Average 2D surface roughness, (b) 2D roughness plots, and (c) 3D surface roughness.

### 3.4 Vickers Hardness of LSPwC AA7075/h-BN Nanocomposites

The Vickers Hardness (HV) results of unpeened and LSPwC AA7075/h-BN MMCs are shown in Figure 9. The average HV of both the unpeened and LSPwC AA7075/h-BN MMCs is reported in Figure 9a and the cross-sectional HV along the depth-wise from the LSPwC region of AA7075/h-BN MMCs is shown in Figure 9b. For unpeened AA7075/h-BN MMCs, the hardness was measured on the finely polished surface at eight different locations with a 100 μm length, and the average was reported. From the figure, it was observed that HV was increased significantly with the inclusion of h-BN NPs and LSPwC. The average hardness for unpeened AA7075 (as-cast) was recorded as 154.4 HV, which increased to 164.5 HV with LSPwC, reflecting an increment of 6.33%. Similarly, the hardness of AA7075/0.5h-BN increased to 7.57%, from 161.3 HV to 174 HV with LSPwC. AA7075/1.0h-BN was improved from 163.7 HV to 185 HV, with an improvement of 12.2% with LSPwC. The presence of h-BN NPs has undoubtedly obtained effective grain refinement and contributed to improving the HV of AA7075. The LSPwC produced an intense plastic deformation leading to high dislocation density, accompanied by effective grain refinement in the surface and subsurface region of AA7075/h-BN composites. Additionally, strain hardening through peening (LSPwC), dislocation densities at the microstructural level, the formation of CRS in the surface and subsurface regions, and grain refinement were primary factors contributing to the improvement of microhardness, as reported by many researchers.^53,57^

**Figure 9:**
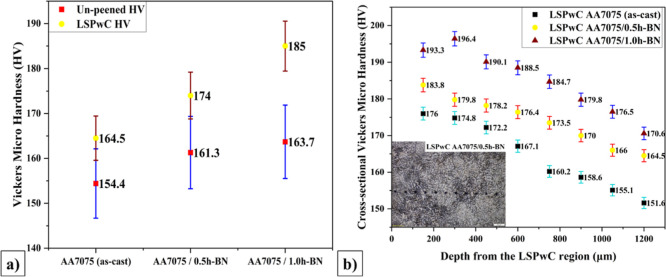
Vickers microhardness of LSP_W_C AA7075/h-BN nanocomposites:(a) average microhardness of un-LSP_W_C and LSP_W_C AA7075/h-BN nanocomposites and (b) depth cross-sectional microhardness variation of LSP_W_C AA7075/h-BN nanocomposites.

### 3.5 Wettability and Surface Energy of LSPwC AA7075/h-BN Nanocomposites

Figure 10 displays the wettability characteristics (contact angle (Φ) and surface energy (Es)) of both unpeened and LSPwC AA7075/1.0h-BN nanocomposites. From Figure 10, an inverse relationship was seen between CA and *E*_s_. As CAs decrease (improved wettability), the *E*_s_ increases; this trend directly reflects the enhanced surface wettability characteristics (solid–liquid interaction). In the unpeened samples, the CA of AA7075 (as-cast) was recorded as 87.75°, which decreased to 84.85° with the inclusion of h-BN NPs in AA7075/0.5h-BN, and further to 79.55° for AA7075/1.0h-BN composites. Correspondingly, *E*_s_ increased from 2.86 mJ/m^2^ to 6.54 mJ/m^2^, and 13.2 mJ/m^2^, respectively. This enhanced wetting behavior was mainly due to the refinement of the microstructure induced by the presence of h-BN NPs in AA7075. The AA7075 as-cast sample has a heterogeneous surface nature (coarse grains with minimal grain boundary activity), which causes poor wettability and minimal surface activity (less spread of liquid), resulting in low *E*_s_. The h-BN NPs contributed to increasing the grain boundary density, improving phase uniformity and increasing surface activity, rendering the surface more hydrophilic. As a result, the DI water (liquid) spreads more effectively across the surface, increasing the contact area and thereby enhancing *E*_s_. Upon LSPwC treatment, a further improvement in the wettability (low CA) and *E*_s_ was observed. For LSPwC AA7075 (as-cast), the CAs reduced drastically from 87.75° (unpeened AA7075 (as-cast) to 77.75° (LSPwC AA7075 (as-cast)) and significantly increased *E*_s_ to 15.45 mJ/m^2^ from 2.86 mJ/m^2^. Similarly, AA7075/h-BN nanocomposite CAs decreased from 84.85° to 77.5° (LSPwC AA7075/0.5h-BN) and 79.55° to 74.25° (LSPwC AA7075/1.0h-BN) and corresponding *E*_s_ increased from 6.54 mJ/m^2^ to 15.45 mJ/m^2^ and 13.2 mJ/m^2^ to 19.76 mJ/m^2^, respectively. The combined effect of h-BN NPs and LSPwC treatment not only improves the microstructure but also promotes better solid–liquid interaction, which accounts for the sharp increase in *E*_s_ and decrease in CA.

**Figure 10:**
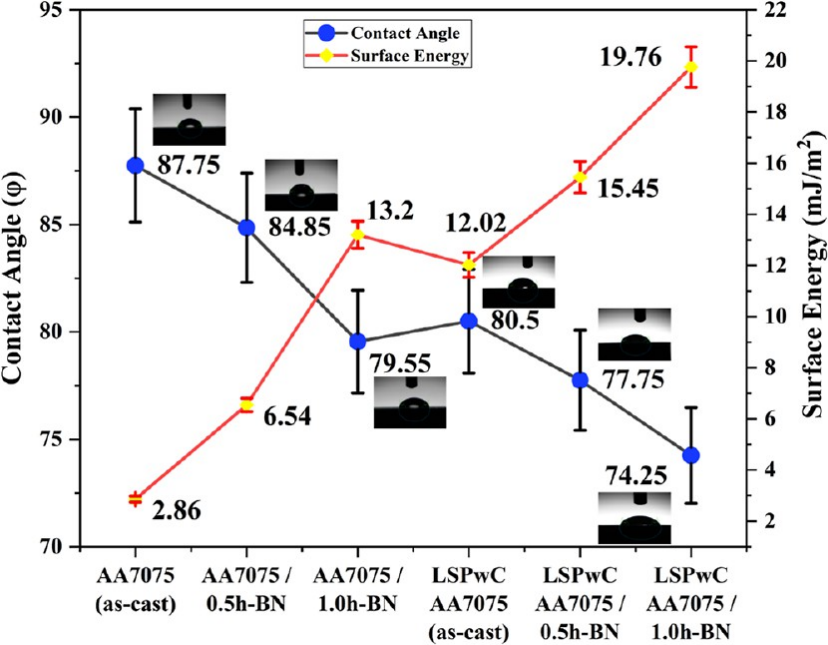
Wetting characteristics of LSP_W_C AA7075/h-BN nanocomposites.

### 3.6 Corrosion Characteristics of LSPwC AA7075/h-BN Nanocomposites

Figure 11 illustrates the corrosion characteristics of both the unpeened and LSPwC AA7075/h-BN nanocomposites. It was quite evident that the inclusion of h-BN NPs improves the corrosion characteristics of both unpeened and LSPwC AA7075/h-BN composites. Also, LSPwC significantly influenced the corrosion properties and reduced corrosion resistance. From the Tafel curves, it was evident that the *E*_corr_ shifts to a more negative value, and *I*_corr_ decreases significantly with the inclusion of h-BN NPs. However, the as-cast AA7075 unpeened specimens exhibited a negative *E*_corr_ of −0.951 and provided the highest *I*_corr_ of ∼7.42 × 10^–8^ A. This results in a lower resistance to corrosion of 0.103 mm/year, which was correlated to a lower LPR of 50829.4 Ω. Additionally, the morphology of the corroded unpeened AA7075 (as-cast) region (shown in Figure 12) exhibits more numerous and larger cracks as well as delamination of corrosion products due to its dendritic structure. This resulted in significant localized corrosion and pitting.

**Figure 11:**
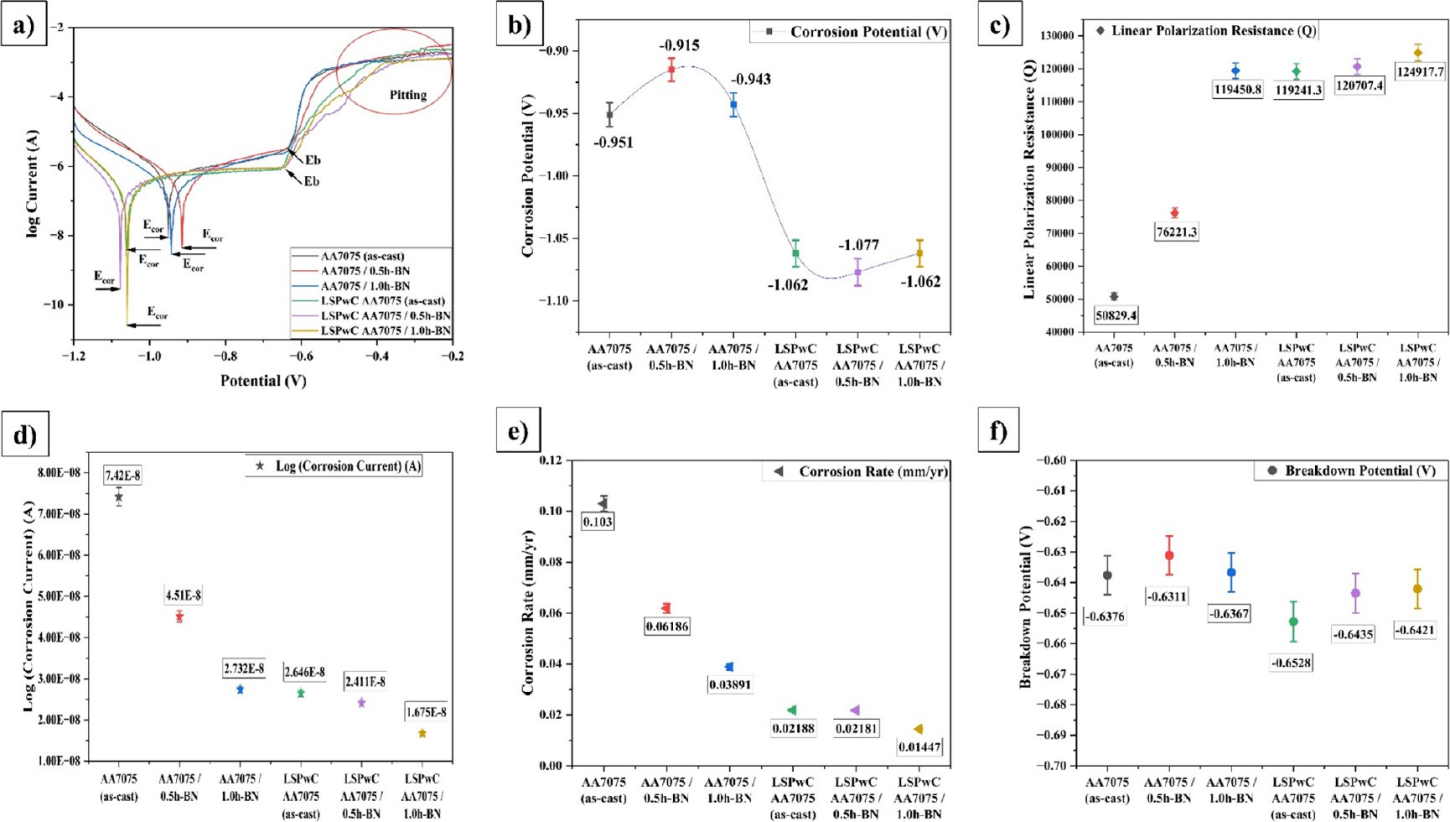
Corrosion characteristics of LSPwC AA7075/h-BN nanocomposites. (a) Potentiodynamic polarization plot, (b) breakdown potential, (c) current potential, (d) corrosion current, (e) linear polarization resistance, and (f) corrosion rate.

**Figure 12:**
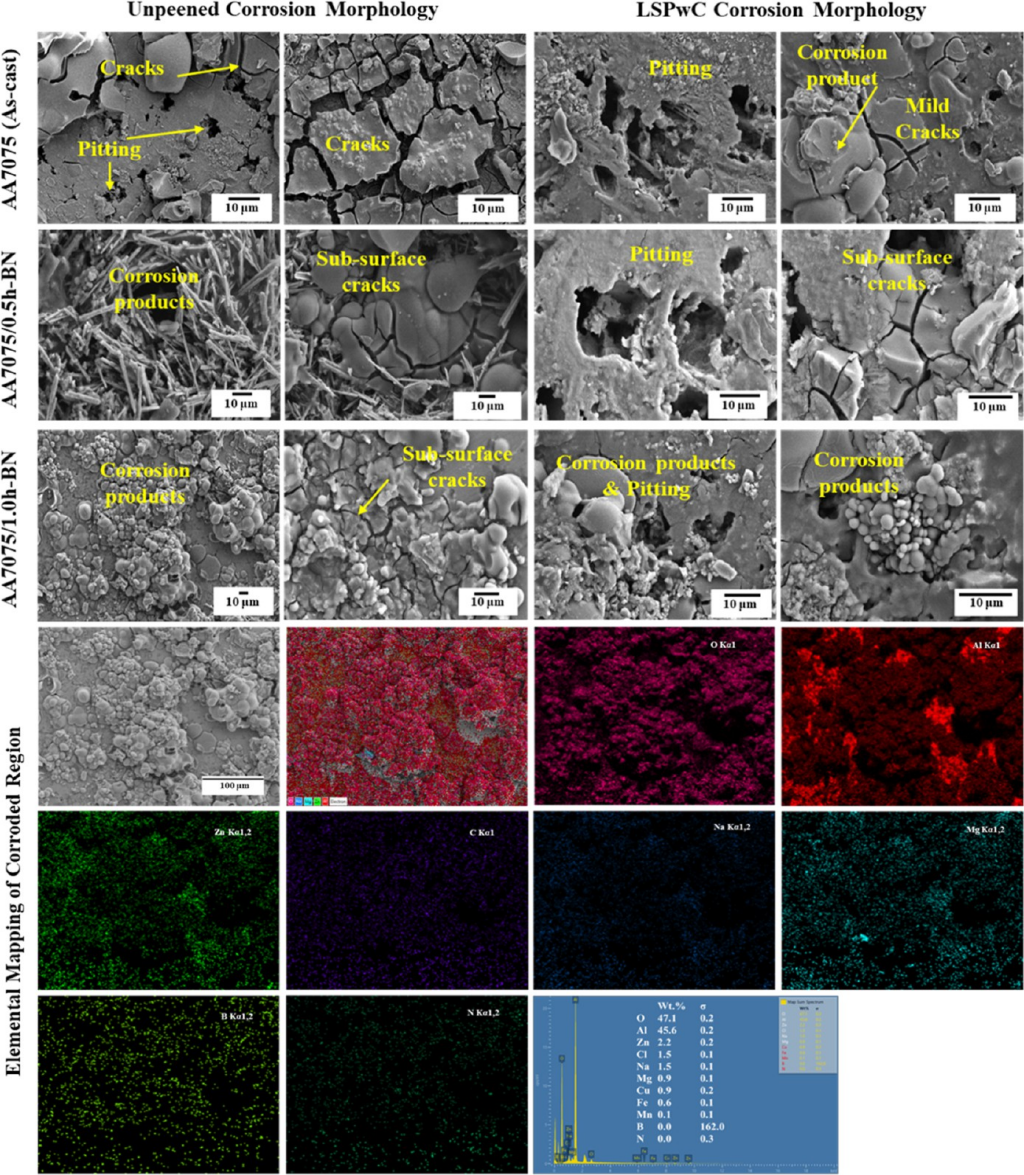
Corrosion morphology of LSPwC AA7075/h-BN nanocomposites.

Incorporation of h-BN NPs in AA7075 improved the corrosion resistance by obtaining a lower CR of 0.06186 mm/year for AA7075/0.5h-BN and 0.03892 mm/year for AA7075/1.0h-BN composites. This resulted in lower *I*_corr_ 4.51 × 10^–8^ A and 2.732 × 10^–8^ A and higher LPR values of 76221.3 and 119450.8 Ω, respectively. The h-BN NPs serve as a barrier because of their hydrophobic and chemically inert nature to resist corrosion. Also, it creates a complex path for corrosion ions, which slows their diffusion toward the Al matrix. Further, the grain boundary pinning with h-BN NPs refined the microstructure and produced more stable passive oxide films like Al_2_O_3_ to resist pitting (localized corrosion) but formed some cracks (ref Figure 12 of AA7075/0.5h-BN). In addition to the above positive features, the further inclusion of h-BN NPs created a stable layer and produced corrosion byproducts (ref Figure 12 of AA7075/1.0h-BN). This will effectively contribute to reducing pitting and overall corrosion.

LSPwC AA7075/h-BN composites showed substantial improvement in corrosion resistance. The LSPwC AA7075 (as-cast) exhibited a reduced corrosion rate of 0.02188 mm/year compared to the as-cast AA7075. Also, the more negative *E*_corr_ value (−1.062 V) was recorded, along with a decreased *I*_corr_ (∼2.646 × 10^–8^ A) and increased LPR of 119241.3 Ω. LSPwC has shown substantial growth in corrosion properties due to the progressive grain refinement and the induction of CRS, as well as surface densification, which suppresses corrosion initiation. These features helped to reduce the cracks effectively. However, some mild pitting was observed beneath the subsurface cracks (ref Figure 12 of LSPwC AA7075).

Notably, h-BN-reinforced AA7075 composites also significantly reduced corrosion resistance after peening. The AA7075/0.5h-BN nanocomposite exhibits a CR of 0.2181 mm/year with a lower *E*_corr_ value of −1.077 V, an *I*_corr_ value of 2.41 × 10^–8^ A, and an LPR of 120707.4 Ω. Further, the AA7075/1.0h-BN nanocomposite exhibits better corrosion resistance with a lower CR of 0.01447 mm/year, minimal *E*_corr_ −1.062 V, least *I*_corr_ 1.675 × 10^–8^ A, and highest LPR 124917.7 Ω. This prominent corrosion resistance is due to the synergistic interaction between the h-BN NPs and LSPwC on AA7075. This allowed the formation of a stable, adherent, and uniform passive oxide film, which minimizes pitting corrosion and provides long-term protection (ref. Figure 12 of LSPwC AA7075/h-BN composites), especially in sacrificial anodic environments.^31,58^ The addition of h-BN NPs enhances the wetting characteristics of both unpeened and LSPwC AA7075/h-BN composites. However, most research has shown that the impact of wetting on corrosion is relatively minor. In this case, the evidence is clear. The main reason for the improved corrosion resistance with the inclusion of h-BN NPs was due to their high electron work function compared to that of the Al matrix. Also, it becomes challenging for electrons to be extracted from the LSPwC AA7075/h-BN composites during the corrosion process to begin since the h-BN NPs are well-distributed on the surface. Therefore, it can be concluded that the addition of an optimal quantity of h-BN increases the corrosion resistance of the AA7075.

### 3.7 Tribological Characteristics of LSPwC AA7075/h-BN Nanocomposites

The SWR and CoF of both unpeened and LSPwC AA7075/h-BN nanocomposites were tested against EN-31 steel at room temperature (Figure 13a) and an elevated temperature of 150 °C (Figure 13b). While the worn-out morphologies are shown in Figure 14. The empty AA7075 (as-cast) sample exhibits the highest SWR of 0.2704 mm^3^/KN-m and a CoF of 0.325, showing poor tribological characteristics among the tested specimens at room temperature. This behavior was attributed to its inherent dendrite grain structure, which resulted in lower hardness and weakened interfacial bonding during sliding. Consequently, these factors led to higher material loss and increased friction. The worn-out morphology of the unpeened AA7075 (as-cast) sample primarily displayed delamination wear alongside severe adhesion of worn-out particles (Figure 14). In comparison, the LSPwC treatment effectively reduced both the SWR and CoF but not significantly. The SWR decreased to 0.2117 mm^3^/KN-m and the CoF reduced to 0.321, respectively. This improvement was mainly due to the induced CRS by LSPwC, which altered the microstructure, enhanced surface hardness, and improved wear resistance. The worn-out morphology of the LSPwC AA7075 (as-cast) sample showed reduced delamination features with some adhesion of worn-out particles.

**Figure 13:**
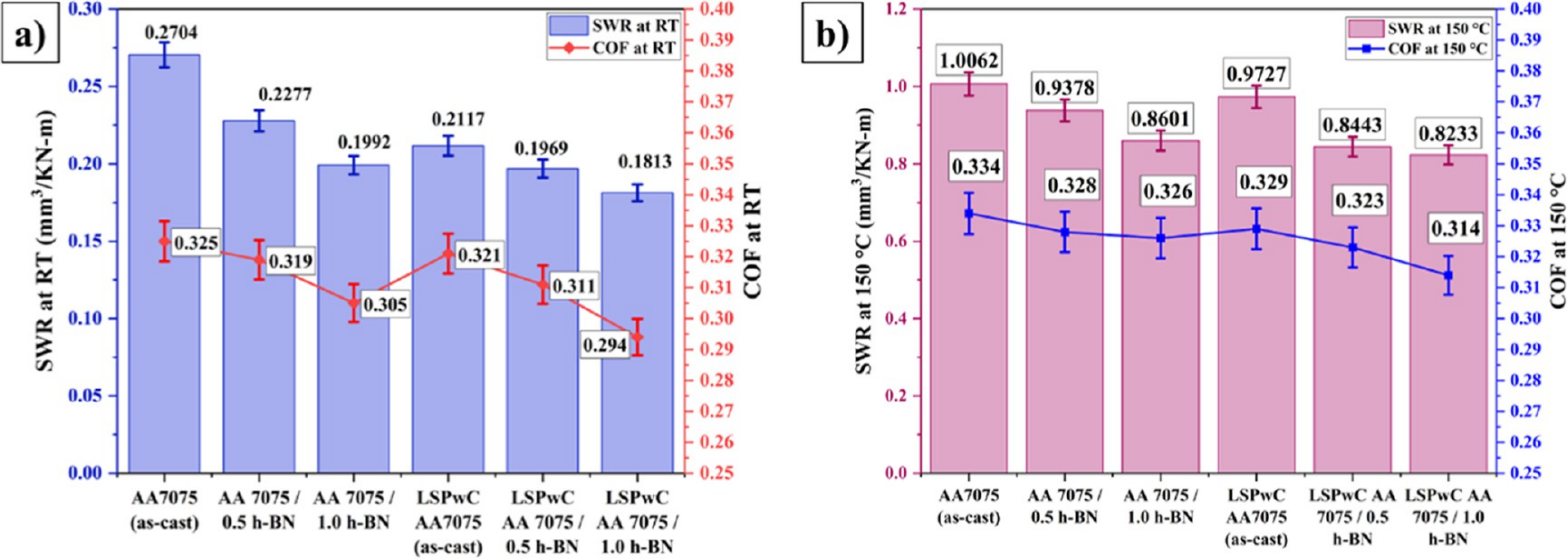
SWR and CoF of unpeened and LSPwC AA7075/h-BN nanocomposites: (a) tested at room temperature (RT) and (b) tested at an elevated temperature of 150 °C.

**Figure 14:**
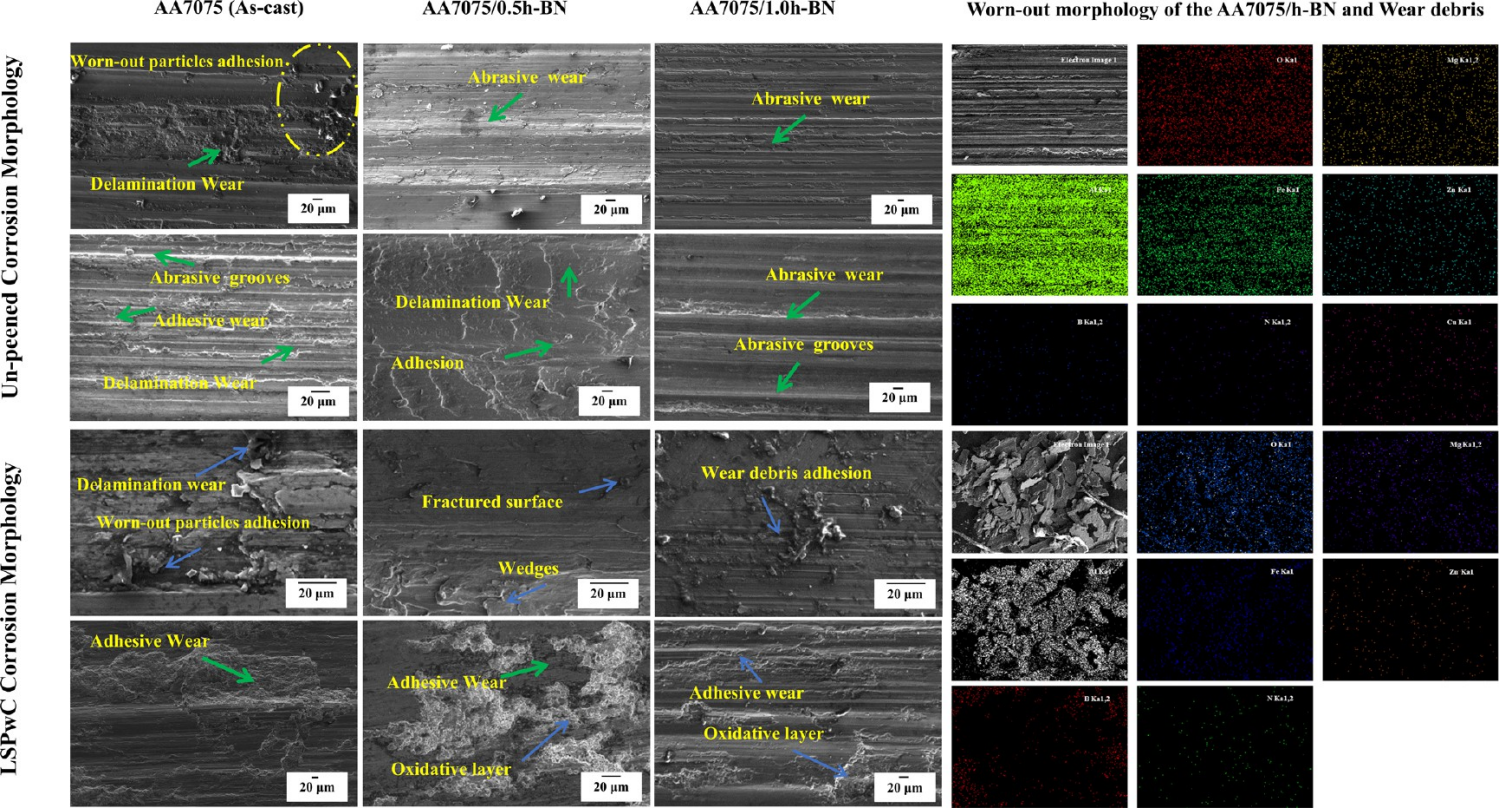
Worn-out morphology of the unpeened and LSPwC AA7075/h-BN nanocomposites at RT and 150 °C.

The inclusion of h-BN NPs significantly influenced both the SWR and CoF under unpeened and LSPwC conditions. For the unpeened AA7075/h-BN composites, incorporating 0.5 and 1.0 wt % of h-BN NPs decreased the SWR to 0.2277 mm^3^/KN-m and 0.1993 mm^3^/KN-m, and the CoF to 0.319 and 0.305, respectively, in comparison to the unpeened AA7075. These improvements were mainly attributed to the presence of self-lubricating h-BN NPs, which enhanced the load-bearing capacity and contact stress of the AA7075. Additionally, the worn-out particles formed a tribo-film (containing h-BN and Al particles), which also contributed to reducing the SWR and CoF. These features helped in the reduction of delamination wear, with predominantly abrasive wear mechanisms and slight debris adhesion.

These benefits were further amplified in the LSPwC-treated AA7075/h-BN composites. The LSPwC AA7075/0.5h-BN and AA7075/1.0h-BN nanocomposites exhibited even lower SWR values of 0.1969 mm^3^/KN-m and 0.1813 mm^3^/KN-m and CoF values of 0.311 and 0.294, respectively. This enhanced tribological performance stemmed from the synergistic effect of surface strengthening caused by LSPwC and the intrinsic lubricating nature of the h-BN NPs. The LSPwC process improved surface hardness, which helped to minimize plastic deformation and microcrack formation during sliding. Simultaneously, the h-BN NPs formed a lubricating layer along with facilitated debris during sliding action, thereby mitigating adhesive and delamination wear. Furthermore, the presence of chemically incompatible metal pairs (Al matrix with h-BN and Zn phases) helped reduce adhesion at the contact interface, further lowering CoF. In conclusion, the enhancement in wear resistance arises from a combination of surface hardening, the solid lubrication effect of h-BN, and interfacial incompatibility with the counter face, making the LSPwC AA7075/1.0h-BN nanocomposite the most wear-resistant among all tested samples at room temperature.

Similarly, the unpeened AA7075 (as-cast) exhibited the highest SWR of 1.0062 mm^3^/KN-m and CoF of 0.334 when tested at 150 °C, compared to other specimens. The worn surface morphology prominently showed abrasive grooves with adhesive and delamination wear characteristics. These features were due to the relatively soft nature of the AA7075 (as-cast) material at this elevated temperature. This softness facilitates easier material removal by hard asperities as well as promotes adhesive interactions and subsurface crack propagation. With the addition of h-BN NPs, both the SWR and CoF decreased even at elevated temperatures (150 °C). The SWR and CoF decreased to 0.9378 mm^3^/KN-m and 0.328, respectively, with the AA7075/0.5h-BN composite. The values further decreased to 8601 mm^3^/KN-m and 0.326, respectively, with AA7075/1.0h-BN nanocomposites. The worn morphology of these samples displayed mild delamination along with adhesive and abrasive wear mechanisms compared with the AA7075 (as-cast). These improvements were mainly attributed to the self-lubricant effect of h-BN particles and the mechanisms discussed above.

Further, LSPwC has significantly decreased the SRW and CoF AA7075/h-BN specimens at elevated temperatures too. LSPwC AA7075 (as-cast) exhibited a lower SWR of 0.9727 mm^3^/KN-m and CoF of 0.29 as compared to the empty AA7075 (as-cast) specimen. Moreover, the excessive delamination and abrasive wear have been converted to adhesive wear with mild delamination features due to the surface hardening achieved through LSPwC. Further, the LSPwC AA7075/0.5h-BN and AA7075/1.0h-BN samples showed significant reductions in SWR to 0.8443 mm^3^/KN-m and 0.8233 mm^3^/KN-m and CoF to 0.323 and 0.314, respectively. The worn-out morphologies of LSPwC AA7075/0.5h-BN and AA7075/1.0h-BN nanocomposites show adhesive wear combined with oxidative wear. During high-temperature wear tests, the oxidation process occurs at the interactive surface, resulting in the formation of an oxidative layer on the worn surface that interacts with h-BN NPs.^5,59,60^ This combined effect (oxidative layer + self-lubricating nature of h-BN NPs), along with the three-body wear mechanisms (wear debris acts as a third body), helps effectively to reduce both friction and wear.

## 4 Discussion on the Effect of LSPwC on Surface Modification of AA7075/h-BN Nanocomposites

LSPwC generates intense ultrahigh-pressure shock waves on the AA7075/h-BN nanocomposite surface. Unlike conventional LSP with an ablative coating, LSPwC functions under thermo-mechanical (combined mechanical shock and localized thermal interaction) conditions,^20^ thereby altering the surface and near-surface characteristics of AA7075/h-BN nanocomposites. Further, the laser–plasma interaction under water confinement generated shock pressures exceeding the HEL, resulting in SPD in the near-surface region.^31^

The LSPwC-treated AA7075/h-BN shows crater-like indentations due to localized melting and rapid resolidification under ultrahigh strain rates, resulting in increased surface roughness compared to the as-cast AA7075/h-BN composites. Although such features increase surface activity, quantitative roughness measurements indicate a ∼10% reduction in *R*_a_ for LSPwC AA7075/1.0h-BN than for LSPwC AA7075 (as-cast). This reduction is attributed to the stabilizing effect of h-BN nanoparticles, which suppresses excessive plastic flow and improves surface uniformity during peening. Importantly, SEM analysis confirms the absence of microcracks because of the controlled laser energy input and pulse overlap.

In addition, the propagation of high-pressure shock waves generated during LSPwC induces a high density of dislocations/rapid dislocation accumulation in the surface and subsurface regions of AA7075/h-BN composites. Further, the combined effect of the high strain rate and localized thermal input suppresses recovery and promotes DRX. Also, it promotes the formation and redistribution of intermetallic precipitates such as MgZn_2_ and Al_2_CuMg within the α-Al matrix. These precipitates preferentially nucleate along LAGBs and dislocation-rich regions. The combined presence of refined grains, precipitates, and CRS contributes significantly to surface hardening. As a direct consequence, LSPwC AA7075/1.0h-BN shows an ∼13% increase in surface hardness compared to unpeened AA7075/1.0h-BN. This clearly links grain refinement and dislocation strengthening to mechanical enhancement.

Electrochemical measurements further confirm the beneficial role of LSPwC in surface modification. The LSPwC AA7075/1.0h-BN nanocomposite shows the lowest corrosion rate among all samples due to the formation of a stable, adherent, and uniform passive oxide film. This minimizes pitting corrosion and provides long-term protection, especially in sacrificial anodic environments. In addition, LSPwC-treated AA7075/1.0h-BN nanocomposite showed a ∼9% and ∼5% reduction in SWR and consistent ∼4% reduction in CoF at RT as well as 150 °C, compared to the unpeened AA7075/1.0h-BN nanocomposite. Significant improvements in the tribological characteristics are due to surface hardening, refined microstructure, and the h-BN’s effective solid-lubricant action. The mechanisms together played a vital role in shifting the wear mechanism from severe abrasion and delamination to mild adhesive wear. The improved surface microstructure by LSPwC directly translates into enhanced hardness, wear resistance, and corrosion performance without altering the bulk composition of AA7075/h-BN nanocomposites.

## 5 Conclusions

In this study, LSPwC was successfully performed on stir-squeeze cast AA7075 and AA7075/h-BN nanocomposites using a Q-switched Nd/YAG laser. The process significantly enhanced the surface morphology, microstructure, mechanical, corrosion, and tribological properties of the LSPwC-treated AA7075/h-BN nanocomposites. The key findings are as follows:

•Post-LSPwC, the AA7075/h-BN nanocomposites exhibited finer grains, better orientation, and DRX-induced texture refinement. Also, intermetallic compounds like AlZn, MgZnAl, and MgAl were observed in the AA7075/h-BN composites.
•The inclusion of h-BN NPs combined with LSPwC (LSPwC AA7075/1.0h-BN) led to an increase in hardness by approximately 13% and a reduction in *R*_a_ by about 10% when compared to LSPwC AA7075 (as-cast).
•Wettability improved significantly with both h-BN addition and LSPwC treatment, indicated by lower CAs and higher SE. This suggests better interaction potential with coatings and working fluids.
•Corrosion performance of the composites improved, especially for LSPwC AA7075/1.0h-BN, which showed the lowest CR (0.01447 mm/year), lowest *I*_corr_ (1.675 × 10^–8^ A), and highest LPR (124917.7 Ω).
•LSPwC AA7075/1.0h-BN nanocomposites demonstrate minimal SWR (0.1813 mm^3^/KN-m at RT and 0.8233 mm^3^/kN-m at 150 °C) and lower CoF (0.294 at RT and 0.314 at 150 °C) among all tested samples. Also, the wear mechanisms shifted from severe abrasion, adhesive wear, and delamination to milder adhesion and delamination in LSPwC-treated composites.

Overall, this study demonstrates that the combined effect of h-BN reinforcement and LSPwC treatment leads to significant improvements in the mechanical, corrosion, and tribological performance of AA7075. This study mainly focused on surface characteristics. In the future, fatigue life evaluation under cyclic loading, depth-resolved residual stress profiling, and high-temperature corrosion characteristics will be investigated for LSPwC-treated AA7075/h-BN nanocomposites.

## Data Availability

All data generated or analyzed during this study are included in this published article (and its Supporting Information files). Code Availability, Ethics Approval, Consent to Participate, Consent for Publication

## Abbreviations

AA: aluminum alloy
Al-MMCs: aluminum metal matrix composites
CA: contact angle
CoF: coefficient of friction
CTE: coefficient of thermal expansion
CRS: compressive residual stress
CR: corrosion resistance
DRX: dynamic recrystallization
*E*_b_: breakdown potential
EDS: energy dispersive spectroscopy
EM: elemental mapping
*E*_s_/SE: surface energy
FE-SEM: field emission-scanning electron microscopy
fwhm: full width at half-maximum
GOS: grain orientation spread
GND: geometrically necessary dislocation
HEL: hugoniot elastic limit
h-BN: hexagonal boron nitride
IMC: intermetallic compounds
IPF: inverse pole figure
IQ: image quality
KAM: karlene average misorientation
LPR: linear polarization resistance
LSP: laser shock peening
LSPwC: laser shock peening without coating
OM: optical microscopy
SEM: scanning electron microscopy
SWR: specific wear rate
SSC: stir-squeeze casting
*R*_a_: surface roughness
VH: vickers hardness
XRD: X-ray diffraction
